# Toward Nonbinary Nuance in Research and Care: Mapping Differences in Gender Affirmation and Transgender Congruence in an Online National U.S. Survey

**DOI:** 10.1089/trgh.2020.0038

**Published:** 2021-06-02

**Authors:** Aaron S. Breslow, Hailey Wojcik, Robert Cox, Nathaniel M. Tran, Melanie E. Brewster

**Affiliations:** ^1^ PRIME Center for Health Equity, Department of Psychiatry and Behavioral Sciences, Albert Einstein College of Medicine/Montefiore Medical Center, Bronx, New York, USA.; ^2^ Health Equity Research Lab, Cambridge Health Alliance, Department of Psychiatry, Harvard Medical School, Boston, Massachusetts, USA.; ^3^ Department of Counseling & Clinical Psychology, Teachers College, Columbia University, New York, New York, USA.

**Keywords:** transgender, nonbinary, congruence, transition, gender affirmation

## Abstract

**Purpose:** To close gaps in transgender health research, we mapped trends in gender affirmation processes (i.e., social, legal, and psychological transitions) that are unique among nonbinary (NB) transgender adults when compared with transgender women (TW) and transgender men (TM).

**Methods:** Data were drawn from the Columbia Trans Empowerment Survey (*N*=707), an online national study conducted between 2014 and 2015 in the United States. We used one-way analysis of variance tests, chi-square tests, Kruskal–Wallis tests, and *post hoc* analyses to estimate differences in gender affirmation processes and transgender congruence between: (1) NB adults, *n*=271, 38%; (2) TW, *n*=291, 41%; and (3) TM, *n*=145, 21%. We then identified bivariate correlations between variables of interest.

**Results:** In the full sample (*n*=707), we found significant positive bivariate correlations between pursuing gender affirmation and transgender congruence. In terms of demographics, NB participants were significantly more likely to be queer (42.1%), polyamorous (25.5%), unemployed (44.8%), and younger (median=22) than TW and TM. They also reported taking significantly fewer gender affirmation processes, with significant differences between the three groups in terms of particular experiences. The NB participants also reported significantly lower rates of transgender congruence, specifically lower appearance congruence though similar gender identity acceptance.

**Conclusion:** The NB transgender adults in this sample report unique identity-related characteristics, including significantly lower rates of medical/social transition as well as decreased transgender congruence. These data are among the first to describe unique pathways by which NB adults, TW, and TM may pursue gender affirmation and interact with providers as they navigate congruence, transition, and well-being.

## Introduction

Recent studies have begun to explore the critical role of gender affirmation processes (i.e., social, medical, and legal steps that people take to actualize one's gender identity) in promoting mental health and mitigating the impact of minority stress among transgender and nonbinary (TNB) adults.^1,2^ For example, TNB adults in a recent study who changed their legal name and/or gender marker reported improved psychological outcomes.^2^ Similar results have evidenced benefits of medical^3^ and social^4^ gender affirmation, including increased transgender congruence (i.e., comfort with one's gender identity and external appearance), and improved psychological outcomes.^5^ For many TNB people, these processes reduce dysphoria and are critical to survival. However, binary, linear models of gender affirmation are limited in their nuance and relevance among growing cohorts of people who identify as nonbinary (NB), for whom disparities in health care access and quality persist.^6–8^

Across TNB samples, approximately one-third identify as NB, an umbrella term describing gender identities that lie between masculine and feminine, hybridize both, or defy restrictive gender ideologies.^9^ For NB individuals, the process of a gender “transition” may be complex, irrelevant, or fraught with misunderstanding from providers.^9–11^ However, many NB people pursue gender affirmation^11^ and may have unique needs compared with transgender women (TW) and transgender men (TM).^12^ Current clinical guidelines poorly reflect such nuances^13,14^ and NB people often report barriers to affirming care, including difficulty finding affirming providers, stigma in health care encounters, and denial of coverage.^11,15,16^ In a 2015 national survey, for example, 70% of NB people reported a need for counseling, yet only 31% accessed care (compared with 73% among TW and TM).^17^ Group disparities likely exist across all gender affirmation processes, though a few data exist regarding capturing unique needs, access, and outcomes.^15,18^

This study fills gaps by mapping unique trends in demographics, gender affirmation processes, and transgender congruence. First, we map differences in demographic characteristics between NB adults, TW, and TM. In recent community surveys, NB people are more likely to identify as queer^19^ and polyamorous^20^ than cisgender people, TW, and TM. However, clinical guidelines rarely attend to intersecting identities and marginalized experiences.^10,21^

Second, we map differences in gender affirmation processes taken by NB participants, TW, and TM.^22^ This line of inquiry is justified given the lack of knowledge about NB-affirming care, including in Version 7 of the World Professional Association for Transgender Health's Standards of Care (SOC). These SOC, though important, do not adequately outline guidelines for gender affirmation processes among NB people, who have often relied on providers who use an “informed consent” rather than a “gatekeeping” model to access care.^9–11^ This study builds on limited knowledge about NB gender affirmation processes to inform future guidelines for TNB care.^23,24^

Third, given associations between gender affirmation and psychological outcomes, we measure differences in transgender congruence. We measured two sub-outcomes: *appearance congruence* (a match between external appearance and internal gendered experience) and *gender identity acceptance* (self-affirmation of transgender identity).^5^ Transgender congruence may differ between NB and binary transgender people, with important implications for care.^15^ Indeed, congruence may not be relevant (or possible) for NB people given the fluidity implicit in NB positionality and limitations in current care options. Recent studies in the United Kingdom^6^ and Canada^25^ demonstrate lower transgender congruence among NB participants, though it remains clear that many NB people wish to pursue medical affirmation and face significant barriers.^6^ To expand on current findings,^26^ we examine differences between NB adults, TW, and TM and provide suggestions for clinical work and research with TNB communities.

## Methods

### Participants and procedures

The TNB adults living in the United States were recruited online for the Columbia Trans Empowerment Study, a national survey focused on marginalization and empowerment among TNB people. Four expert reviewers (e.g., leading members of TNB communities) provided feedback to ensure protocols were affirming^22,23^ and congruent with guidelines for online TNB health research.^24^ The study received research ethics committee approval from the Teachers College, Columbia University Institutional Review Board. Participants were recruited on TNB social media pages and listservs for community centers, university groups, and political organizations between 2014 and 2015. Interested participants were directed to an online study hosted on Qualitrics.com and provided information about rights, risks, and benefits. Participants clicked Yes/No to confirm eligibility (identifying as TNB, 18+ years old, U.S.-based) and to provide informed consent.

In total, 1485 individuals clicked the link; 2 declined to consent. Of the remaining 1483, 572 consented though they answered no additional items. The remaining 911 were screened for eligibility; 73 were excluded due to age (*n*=47) or location (*n*=26). Of the remaining 838, 107 completed <80% of 110 items and were removed. Eight were removed for failing >1 validity check (e.g., “Please select ‘Somewhat Disagree’”). Sixteen were removed for falsified/duplicate data. The final sample consisted of 707 U.S. TNB adults.

### Measures

#### Demographic characteristics

Participants indicated their gender, race, residential environment, sexual orientation, relationship status, employment, education, socioeconomic class, HIV testing and status, and TNB community connectedness (Table 1).

**Table 1.** Demographic Characteristics of Full Sample and Differences by Subgroup

Demographic variables	Total (*n*=707)		Group a, nonbinary (*n*=271)		Group b, transgender women (*n*=291)		Group c, transgender men (*n*=145)		χ^2^	df	*p*	Post hoc
	*n*	%	*n*	%	*n*	%	*n*	%				
Race									13.711	12	0.320	—
Asian-American	26	3.9	12	4.6	7	5.0	7	2.6				
Black/African American	24	3.6	7	2.7	5	3.6	12	4.4				
Latinx American	27	4.0	11	4.2	8	5.7	8	2.9				
Native American	7	1.0	2	0.8	3	2.1	2	0.7				
White	505	75.0	188	72.0	102	72.9	215	79.0				
Multiracial or Biracial	74	11.0	34	13.0	14	10.0	26	9.6				
Other	10	1.5	7	2.7	1	0.7	2	0.7				
Environment									2.923	4	0.571	—
Urban	286	42.6	115	44.2	54	38.8	117	43.0				
Suburban	282	42.0	111	42.7	63	45.3	108	39.7				
Rural	103	15.4	34	13.1	22	15.8	47	17.3				
Sexual orientation									164.565^a^	10	<0.001	a ≠ b, c
Gay/lesbian	98	14.6	19	7.3	46	33.1	33	12.1				
Queer	204	30.4	110	42.1	12	8.6	82	30.1				
Pansexual	102	15.2	43	16.5	20	14.4	39	14.3				
Bisexual	86	12.8	27	10.3	26	18.7	33	12.1				
Heterosexual/straight	106	15.8	8	3.1	25	18.0	73	26.8				
Other	76	11.2	54	20.7	10	7.2	12	4.4				
Relationship orientation									27.173^a^	2	<0.001	a ≠ b, c
Monogamous	590	83.5	202	74.5	124	85.5	264	90.7				
Polyamorous	117	16.5	69	25.5	21	14.5	27	9.3				
Employment status									11.882^b^	4	0.018	a ≠ b, c
Full time	213	31.8	65	24.9	54	38.8	94	34.9				
Part time	196	29.3	79	30.3	35	25.2	82	30.5				
Unemployed	260	38.9	117	44.8	50	36.0	93	35.6				
Education									7.184	6	0.304	—
Some high school or less	21	3.1	11	4.2	3	2.2	7	2.6				
High school diploma	79	11.8	33	12.6	9	6.5	37	13.6				
Undergraduate	446	66.4	166	63.6	100	71.9	180	66.2				
Graduate	126	18.7	51	19.5	27	19.4	48	17.6				
Social class									0.516	4	0.972	—
Upper/upper-middle	102	15.2	41	15.8	21	15.1	40	14.8				
Middle class	261	39.0	97	37.3	56	40.3	108	39.9				
Working/poor	307	45.8	122	46.9	62	44.6	123	45.4				
Lifetime history of HIV test									4.829	10	0.902	—
No	292	43.5	119	46.1	55	39.3	118	43.2				
Yes	379	56.5	152	53.9	85	60.7	155	56.8				
Community connectedness									5.711	8	0.680	—
Not at all	40	6.0	14	5.4	7	5.0	19	7.0				
Very little	174	25.9	63	24.1	32	23.0	79	29.0				
Moderately	238	35.4	101	38.7	47	33.8	90	33.1				
Quite a bit	145	21.6	56	21.5	34	24.5	55	20.2				
Extremely	75	11.2	27	10.3	19	13.7	29	10.7				
Age, median (IQR)	26 (20–23)		22 (19–27)		27 (21–42)		23 (20–27)		35.625^a,c^	2	<0.001	a ≠ b, c

Total *n*=707. Some values do not add to 707 or 100% due to either missing information or “select all that apply.” *Post hoc*: As appropriate, *post hoc* tests were conducted by using partial χ^2^ or Mann–Whitney U with LSD criterion.

^a^ *p*<0.001.

^b^ *p*<0.05.

^c^ Kruskal–Wallis test (nonparametric equivalent to ANOVA).

ANOVA, analysis of variance; df, degrees of freedom; IQR, interquartile range; LSD, lysergic acid diethylamide.

#### Gender affirmation processes

Participants answered the following question: “Have you ever taken any of these steps to change how you present your gender to others?” by clicking Yes/No to indicate if they had: (1) changed their name, (2) changed their hair, (3) changed the sex on their ID, (4) changed their clothes, (5) changed their face with surgery, (6) changed the structure of their neck, (7) changed their chest, (8) had bottom surgery, (9) used hormone therapy treatment, or (10) changed their appearance in other ways. The total number of “Yes” items were summed to create a composite score. Participants who changed their appearance in other ways (*n*=216) explained further in a free-text box. Qualitative data were blind-coded by the two first authors who met to resolve discrepancies.

#### Transgender congruence

Participants completed the 12-item Transgender Congruence Scale^5^ to report the extent to which they felt genuine, authentic, and comfortable with their gender identities and personal appearance (e.g., “My outward appearance represents my gender identity,” “I feel that my mind and body are consistent with one another.”) Item scores were averaged; higher scores indicate higher transgender congruence.

### Analysis plan

Analyses were conducted in SPSS. First, we conducted analyses of variance (ANOVAs), chi-square tests (*α*=0.05), and Fisher's exact tests (when expected cell counts <5) to compare group differences by outcome. Second, when group differences were significant, we conducted Kruskal–Wallis tests to determine difference distributions by group. *Post hoc* tests included partial chi-square and Mann–Whitney U with lysergic acid diethylamide (LSD) criterion. Third, we determined bivariate correlations via Pearson correlations.

## Results

### Demographic characteristics

In the full sample, participants most frequently identified as: TW (41.2%); White (75%); urban (42.6%); queer (30.4%); monogamous (83.5%); unemployed (36.8%); started/completed undergrad (63.1%); and middle class (36.9%). Just more than half (56.5%) received an HIV test, with 98.4% who had received a negative result. Participants most frequently reported moderate TNB community connectedness (35.4%). Age: 18–71 years old (M=26.31, median=23, standard deviation [SD]=9.82).

Gender identity (NB, TW, TM) yielded nonsignificant associations with race, environment, education, class, HIV testing and status, and community connectedness. There were some significant differences. In particular, NB participants were significantly more likely to identify as queer (*χ*^2^=164.565, degrees of freedom [df]=10, *p*<0.001), polyamorous (*χ*^2^=27.173, df=2, *p*<0.001), and unemployed (χ^2^=11.882, df=4, *p*=0.018) compared with TW and TM despite similar levels of education. The NB participants were significantly younger (median=22, interquartile range [IQR]: 19–27) than TW (median=27, IQR: 21–42) and TM (median=23, IQR=20–27; *χ*^2^=35.625, df=2, *p*<0.001).

### Gender affirmation processes

See Table 2 for gender affirmation processes by full sample and subgroup. In the full sample (*n*=707), participants most frequently (23.6%) endorsed at least 3 out of 10 processes (M=3.79, SD=1.83, range 0–10). For exploratory analysis, we used a chi-square test to compare NB participants with a combined “binary” group composed of all the TW and the TM in the sample. Between these two groups, the “binary” group had taken more steps to affirm their gender (*χ*^2^=81.963, df=10, *p*<0.001). We then compared differences between the three groups. A one-way ANOVA indicated significant differences (*F*=22.90, df=2, *p*<0.001), with NB participants reporting significantly fewer processes than TW (mean difference=−0.95, *p*<0.001) and TM (mean difference=−0.92, *p*<0.001). The TW and TM reported a similar count of total gender affirmation processes.

**Table 2.** Gender Affirmation Processes for Full Sample and Differences by Subgroup

Gender affirmation process	Full sample (*n*=707)		Group a, nonbinary (*n*=271)		Group b, transgender women (*n*=291)		Group c, transgender men (*n*=145)		χ^2^	df	*p*	Post hoc
	*n*	%	*n*	%	*n*	%	*n*	%				
Changed your name									13.365^a^	2	0.001	a ≠ c
Yes	465	65.8	159	58.7	93	64.1	213	73.2				
No	242	34.2	112	41.3	52	35.9	78	26.8				
Changed your hair									3.148	2	0.21	—
Yes	594	84.0	236	87.1	120	82.8	238	81.8				
No	113	16.0	35	12.9	25	17.2	53	18.2				
Changed the sex on your ID									60.865^b^	2	<0.001	a ≠ b ≠ c
Yes	200	23.8	33	12.2	46	31.7	121	41.6				
No	507	76.2	238	87.8	99	68.3	170	58.4				
Changed the clothes you wear									6.874^c^	2	0.03	b ≠ c
Yes	606	85.7	236	87.1	131	90.3	238	81.8				
No	101	14.3	35	12.9	14	9.7	53	18.2				
Changed your face with surgery									N/A^a^	2	<0.001	b ≠ a, c
Yes	12	1.7	1	0.4	9	0.4	2	0.7				
No	695	98.3	270	99.6	136	93.8	289	99.3				
Changed the structure of your neck									3.082	2	0.21	—
Yes	27	3.8	6	2.2	138	95.2	14	4.8				
No	680	96.2	265	97.8	7	4.8	277	95.2				
Changed your chest									57.865^b^	2	<0.001	c ≠ a, b
Yes	163	23.1	36	13.3	18	12.4	109	37.5				
No	544	76.9	235	86.7	127	87.6	182	62.5				
Had genital surgery									21.593^b^	2	<0.001	a ≠ b ≠ c
Yes	33	4.7	5	1.8	17	11.7	11	3.8				
No	674	95.3	266	98.2	128	88.3	280	96.2				
Used hormone therapy treatment									136.409^b^	2	<0.001	a ≠ b ≠ c
Yes	365	51.6	66	24.4	111	76.6	188	64.6				
No	342	48.4	205	75.6	34	23.4	103	35.4				
Changed your appearance in other ways									9.835^a^	2	0.007	c ≠ a, b
Yes	213	30.1	92	33.9	52	35.9	69	23.7				
No	494	69.9	179	66.1	93	64.1	222	76.3				

Total *n*=707. Some values do not add to 707 or 100% due to either missing data or “select all that apply.” For “changed your face with surgery,” a Fisher's exact test was used due to cell counts falling <5. a: Denotes Fisher's exact test performed due to cell value <5. *Post hoc*: As appropriate, *post hoc* tests were conducted by using partial χ^2^ or Mann–Whitney U with LSD criterion.

^a^ *p*<0.01.

^b^ *p*<0.001.

^c^ *p*<0.05.

The three groups reported significant differences in terms of eight gender affirmation processes. First, NB participants were significantly less likely than TM to have changed their name (58.7% compared with 73.2%, χ^2^=13.365, df=2, *p*<0.001), with no significant differences when compared with TW. Second, NB participants were significantly less likely than TM and TW to have changed the sex on their ID (χ^2^=60.865, df=2, *p*<0.001). Third, TW were significantly more likely than TM to have changed the clothes they wear (χ^2^=6.874, df=2, *p*=0.03), with nonsignificant differences when compared with NB participants. Fourth, in terms of changing one's face with surgery, Fisher's exact test revealed significant differences between TW (0.4%), TM (0.7%), and NB (0.4%) participants. Fifth, TM were significantly more likely than NB participants and TW to have changed their chest (χ^2^=57.865, df=2, *p*<0.001), though differences between NB participants and TW were nonsignificant. Sixth, in terms of genital (or “bottom”) surgery, *post hoc* tests revealed significant differences between all three groups (χ^2^=21.593, df=2, *p*<0.001). The TW were the most likely to have had genital surgery (11.7%), with ∼3.8% of TM and 1.8% of NB participants having done so. Seventh, all three groups differed in terms of hormone therapy treatment. The TW reported the highest rates (76.6%), compared with TM (64.6%) and NB participants (24.4%; χ^2^=136.409, df=2, *p*<0.001). Eighth, NB participants and TW were significantly more likely than TM to have changed their appearance in other ways, with nonsignificant differences between NB and TW participants. There were no significant group differences in terms of who was more likely to have changed their hair or changed the structure of their neck.

We also analyzed qualitative data provided by participants about other ways they had pursued gender affirmation. The following themes and frequencies emerged: *changed facial or body hair* (16.4%); *binding* (14.6%); *makeup* (11.3%); *packing* (6.1%); *voice change* (5.6%); *body language/mannerisms* (4.7%); *diet or exercise* (3.8%); *tattoos or piercings* (3.3%); *packing or shapewear* (1.4%); *tucking* (0.5%); and *pronouns* (0.5%). Sample sizes were too small to reveal group differences in terms of having changed one's appearance in these other ways.

### Transgender congruence

In the full sample, the mean transgender congruence score was 3.01 (*α*=0.91, SD=0.93, range 1–5). Subscale scores were: 2.73 for Appearance Congruence (*α*=0.94, SD=1.13, range 1–5) and 3.93 for Gender Identity Acceptance (*α*=0.72, SD=0.90, range 1–5). In terms of subgroup differences, NB participants reported significantly lower transgender congruence than TW and TM. Mean rates of transgender congruence (range 1–5) were 3.01 (SD=0.93) for the full sample; 3.23 (SD=1) for TM; 3.17 (SD=1) for TW; and 2.74 (SD=0.73) for NB participants. Analyses revealed significant overall group differences (*F*=22.93, df=2, *p*<0.001), with NB participants reporting significantly lower scores than TW (mean difference=−0.43, *p*<0.001) and TM (mean difference=−0.49, *p*<0.001). The TW and TM, however, reported similar rates of transgender congruence.

To explore this further, we compared subscales between the three groups. The groups had significantly different scores on the appearance congruence subscale (range 1–5). Mean rates were 2.73 (SD=1.13) for the full sample, 3.01 (SD=1.21) for TM, 2.89 (SD=1.19) for TW, and 2.34 (SD=0.86) for NB participants. In *post hoc* analyses, NB participants reported significantly lower appearance congruence scores than TM (mean difference=−0.67, *p*<0.001) and TW (mean difference=−0.54, *p*<0.001). Differences between TM and TW were nonsignificant. Group differences were nonsignificant for the gender identity acceptance subscale.

### Bivariate correlations

See Table 3 for full-sample bivariate correlations and descriptive statistics. All variables were significantly positively associated at the *p*<0.01 or <0.001 with the exception of the Gender Identity Acceptance Subscale.

**Table 3.** Bivariate Correlations, Descriptive Statistics, and Chronbach's Alpha for Variables of Interest

Variable	(1)	(2)	(2a)	(2b)	Possible range	M	SD	α
(1) Gender Affirmation Processes	—				0–10	3.79	1.83	—
(2) Transgender Congruence	0.36^a^	—			1–5	3.01	0.93	0.91
(2a) Appearance Congruence Subscale	0.38^a^	0.97^a^	—		1–5	2.73	1.13	0.94
(2b) Gender Identity Acceptance Subscale	0.04	0.49^a^	0.27^a^	—	1–4	3.93	0.90	0.72

^a^ *p*<0.001.

SD, standard deviation.

## Discussion

In this online US sample, NB participants reported fewer gender affirmation processes and lower transgender congruence than TW and TM. They also were more likely to identify as queer, polyamorous, young, and unemployed. Across subgroups, we found a positive, significant association between gender affirmation and transgender congruence, though only in terms of appearance and not in terms of gender identity acceptance. These results build on findings from prior research^26^ documenting group-level differences within TNB communities that are crucial for care and collaborative research.

Demographic findings were consistent with prior research.^27^ These results support recent studies demonstrating that NB people may embrace fluidity and reject binarization beyond their gendered experiences. Being queer- and polyamory-affirming is, thus, critical for NB-competent care. Another notable difference was a higher rate of unemployment for NB participants, even with similar education levels. The NB participants were younger than TW and TM and thus perhaps more likely to be in school and/or not yet in the workforce, yet these results echo recent calls for NB-affirming employment pipelines and nondiscrimination policies.^27^

Regarding gender affirmation processes, TW and TM took significantly more steps than NB participants. These findings support studies demonstrating that NB people may not need to undergo social, legal, or medical changes to affirm their gender.^9^ Similarly, they may reflect disparities in access to care and available medical interventions between NB people, TW, and TM. The very concept of transition may be irrelevant or marginalizing in its binary frame, yet NB people continue to report a burden of unmet need for gender-affirming care.^28^

Major differences also emerged between TM and TW. The TM were more likely to legally change their names/gender markers and to have top surgery. These differences may highlight inequities in availability of safe, validated gender affirmation procedures and/or legal/social resources.^6^ The TW were more likely than TM to have had “bottom” surgery and use hormone treatment, perhaps as a result of advancements in vaginoplasty compared with phalloplasty^12^ as well as the gendered ways TW^29^ and TM^30^ contend with sexual objectification. In terms of “top” surgery, TM may be more likely to pursue this process than TW because breast growth can often be achieved through hormone therapy.^18^

In terms of transgender congruence, NB participants reported significantly lower scores than TM and TW. This implies that the concept of “congruence” is not relevant for NB people and/or the lack of NB-affirming options persists. Similarly, the (outdated) notion that TNB people aim to “pass” in some “congruent” way may not be relevant for NB people. This is perhaps best understood by our finding of nonsignificant differences in terms of gender identity acceptance, yet lower appearance congruence for NB participants, who accept their gender identity despite their gendered appearance not being congruent. Given that this study was cross-sectional, we cannot conclude whether NB participants were first not affirmed and subsequently experienced lower congruence, or whether NB people simply defy binary notions of congruence. Recent studies have demonstrated both.^2,28^ Either way, these findings reflect emerging self-definition and creative gender expression in NB communities.

### Limitations

Findings must be interpreted in light of a number of limitations: sample demographics (i.e., majority White, young, and urban); cross-sectional design, calling for longitudinal studies; and the creation of a false trichotomy (NB, TW, and TM) by which TNB people were compared. We are unable to report on differences in gender affirmation processes by sex assigned at birth. Per feedback from NB expert reviewers, we chose not to ask nor report on sex assigned at birth to avoid invalidating TNB people by categorizing participants by “biology” or social/medical labels.^31,32^ We stand by this approach while acknowledging its limitations. First, we are unable to report on differences in outcome by sex assigned at birth; as a result, we cannot make claims about utilization of particular types of hormone/surgical intervention that are specific to bodily difference. Second, there is debate around which aspects of TNB people's lives to capture in health research. Many scholars recommend using a two-step method that captures sex assigned at birth and current gender identity.^29,30^ In future studies, we recommend researchers more rigorously capture details of gender affirmation processes by capturing differences by group in terms of: (1) desire for intervention, (2) utilization of intervention, and (3) sociocultural determinants of health and access. These outcomes can be assessed by differences in sex assigned at birth, gender identity, or both depending on the aims and goals of each study.^33^ Similarly, future studies may wish to conduct further analysis of the differences between NB and “binary” (i.e., TW and TM) people. This may reduce limitations in terms of differential access to certain interventions.

Our sample consists of NB people who indicated that they identify as transgender, thus limiting generalizability to people who identify as NB and as transgender (many NB people do not identify as transgender).^34^ We also encourage future studies to examine disparities in affirmation processes by race, gender, and social class and to examine ways that NB people may engage in social transition not captured in this survey.^11,14^ Finally, transgender congruence may differ significantly for individuals during the gender affirmation process.^11^ We recommend future studies compare nuances in congruence by stage, identity, and desired outcomes.

These findings call for more nuanced care with NB people. Mental health professionals working with NB individuals should work to bolster gender affirmation by engaging in care that affirms flexibility and fluidity in gender, rather than endorsing a transition as healthy or even normative.^10,35,36^ They also call for more nuanced research processes. The Transgender Congruence Scale^5^ may have been developed with TW and TM in mind; we encourage psychometric development of NB-affirming measures and the inclusion of NB people in study design and implementation.^31^ We also recommend that future studies incorporate intersectional approaches (framing intersections as key predictors) by capturing the impact of structural racism, ageism, and disparities in access on gender affirmation outcomes.^37^

## Conclusion

Our findings are in line with recent studies demonstrating transition- and health-related differences between NB adults, TW, and TM.^6,17^ Further inquiry is critical given that NB adults report deleterious health outcomes due to minority stressors and will benefit from resilience-bolstering, NB-affirming interventions.^10,38^ Despite the need, NB individuals are likely to avoid care and/or not return. Indeed, if care competencies ubiquitously encourage bolstering transgender congruence and facilitating transition,^6^ guidelines need to be amended to support other markers of positive outcomes^10^ We hope these findings expand on recent efforts to measure and mitigate NB health disparities, including interventions to facilitate affirmation, decrease stigma, and promote health and health care for all TNB people.

## Abbreviations Used

ANOVA: analysis of variance
df: degrees of freedom
IQR: interquartile range
NB: nonbinary
SD: standard deviation
SOC: Standards of Care
TM: transgender men
TNB: transgender and nonbinary
TW: transgender women
